# Intrasphenoidal Internal Carotid Artery Aneurysm: Rare Cause of Recurrent Epistaxis

**DOI:** 10.22038/ijorl.2025.82770.3788

**Published:** 2025

**Authors:** Harneet Narula, Aditi Vohra, Gavinder Singh Bindra, Amit Jain

**Affiliations:** 1 *Department of Radiodiagnosis, MMIMSR Mullana, * *Ambala,* * Haryana, India.*; 2 *Department of Neurosurgery, MMIMSR Mullana, * *Ambala,* * Haryana, India.*

**Keywords:** Aneurysm, Epistaxis, Internal carotid artery, Sphenoid sinus

## Abstract

**Introduction:**

Epistaxis is a common Otorhinolaryngological condition that is usually self-limiting and rarely requires imaging and intervention. Intrasinus Internal Carotid Artery aneurysm presenting with epistaxis is extremely rare.

**Case Report:**

We report a case of a 57- year old male presenting with recurrent episodes of epistaxis. Contrast-enhanced Computed Tomography of the Paranasal Sinuses and Magnetic Resonance Imaging were performed, which showed a focal defect in the posterolateral wall of the sphenoid sinus through which an aneurysm from the cavernous segment of the Internal Carotid artery was seen herniating into the sinus with an associated hematoma.

**Conclusion:**

Non-traumatic Internal carotid artery Intrasphenoidal aneurysms are infrequent causes of recurrent epistaxis. They can mimic malignant or inflammatory sinus masses, which can lead to inadvertent biopsy; hence, they should be ruled out by radiological investigation to ensure timely diagnosis and management.

## Introduction

Epistaxis is a common self-limiting medical condition that is mostly idiopathic. The common causes of epistaxis in adults include dryness of the nasal mucosa, local trauma, hypertension, coagulation disorders, etc. while Sino-nasal masses are among the rare causes (1). ICA aneurysms are one of the rarer causes of recurrent/massive epistaxis, most commonly being post-traumatic, while non-traumatic true ICA aneurysms are the rarest. 

We report a rare case of non-traumatic ICA aneurysm with intrasphenoidal extension presenting with recurrent epistaxis.

## Case Report

A 57- year old male presented to the ENT department with a nasal bleed from the right nostril. He had a history of recurrent epistaxis for 5 months, which was resolved by nasal compression given by the patient at home. There was no history of hypertension, previous trauma, blood dyscrasias, etc. Vitals were stable. Haemoglobin level was 9.6g/dl. BP was 140/88mm Hg. The coagulation profile was normal. In the present episode, blood loss was approximately 200ml. 

**Fig 1:**
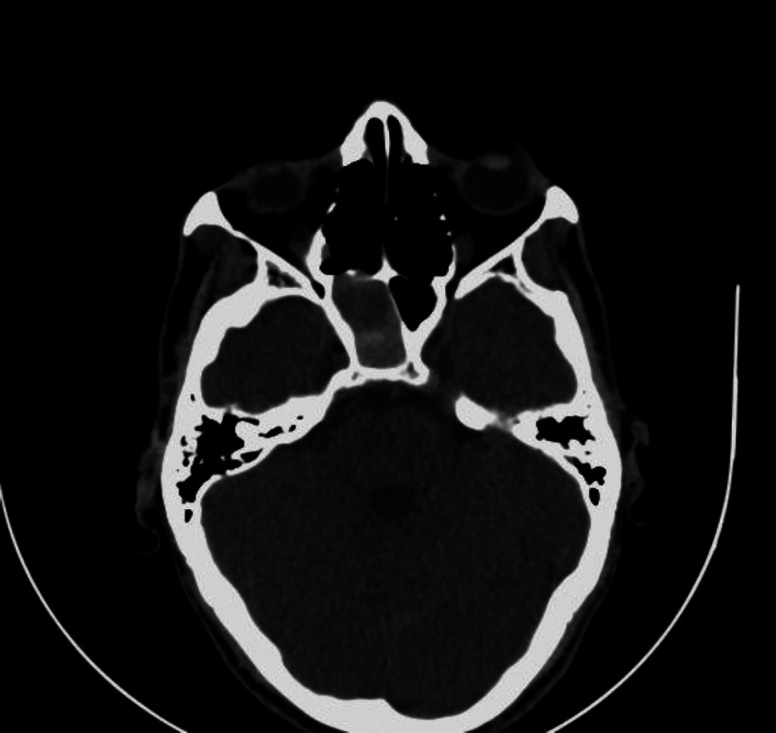
NCCT shows hyperdense contents within the sphenoid sinus

Anterior Rhinoscopy was normal. The patient was managed conservatively by medical treatment and nasal packing. The patient was referred to our department for a CT scan of the Paranasal Sinuses to evaluate the cause of recurrent epistaxis. A non-contrast CT PNS was performed, which revealed hyperdense contents within the sphenoidal sinus (Figure 1).

A focal defect was seen in the posterosuperior wall of the sphenoid sinus on the right side. This was followed by CECT and MRI for further evaluation. 

**Fig 2:**
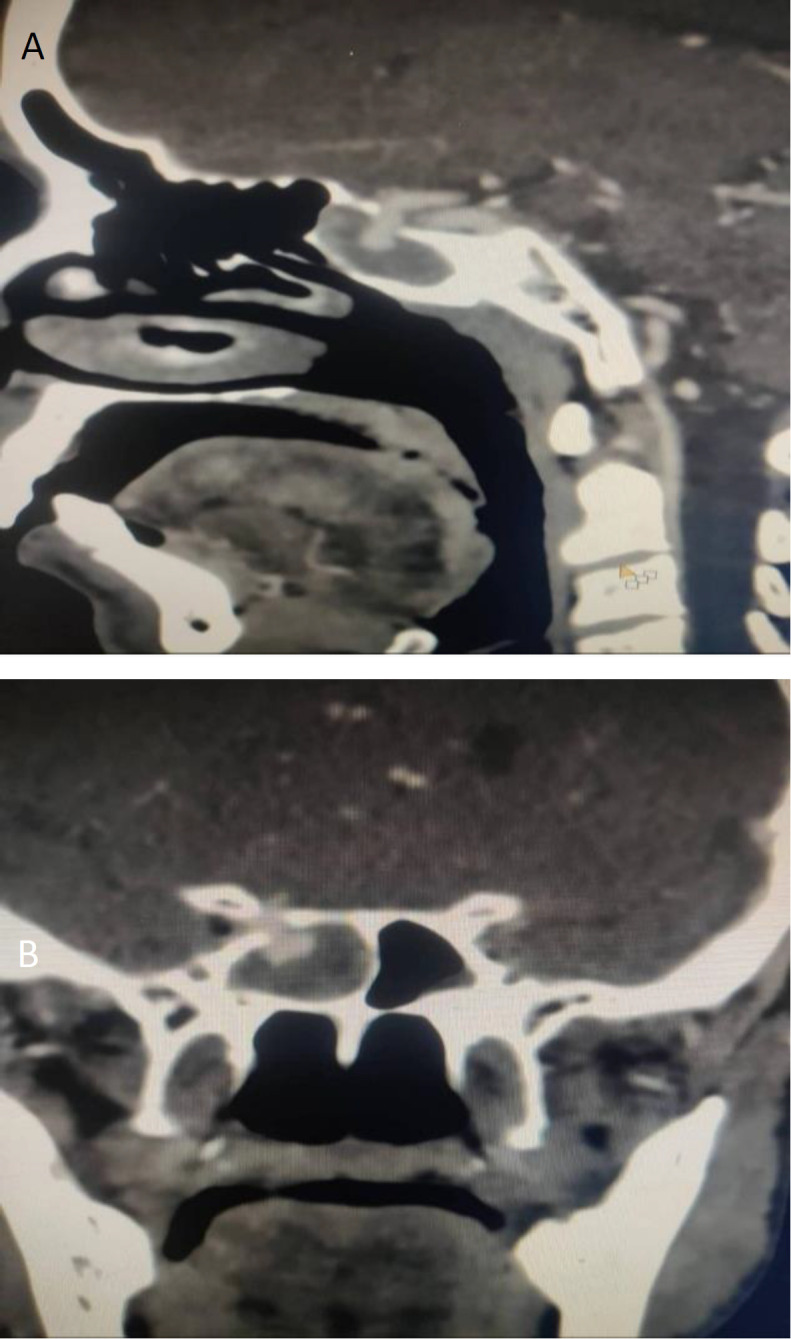
Sagittal (2A) and Coronal (2B) CECT PNS shows an aneurysm of the cavernous segment of right ICA herniating into the sphenoid sinus through a bone defect in the posterolateral wall of the sinus.

CECT revealed a saccular aneurysm of size 7*5mm, with a neck of size 2.4mm, arising from the cavernous segment of right ICA and projecting within the sphenoid sinus on the right side through the bony defect of size 3.9*5.9mm in the sphenoid sinus wall (Figure 2). 

The aneurysm showed irregular walls, suggesting its rupture, and was surrounded by hyperdense hematoma. No obvious active extravasation of contrast was seen. The rest of the paranasal sinuses and nasal cavity were unremarkable.

**Fig 3:**
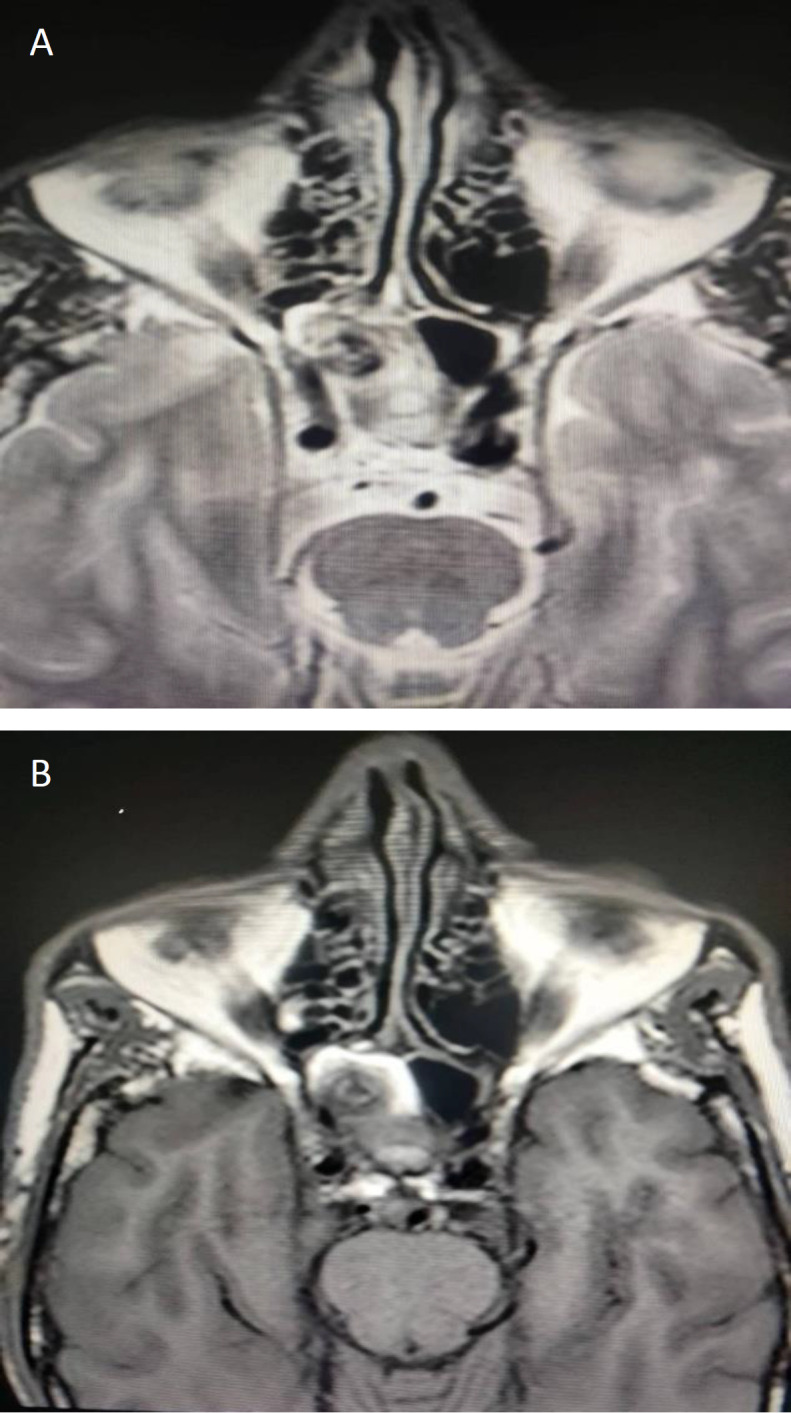
T2W (3A) and T1W (3B) axial MRI scans show a rounded T2 signal void within the sphenoid sinus in continuity with the cavernous segment of right ICA, suggestive of an aneurysm, surrounded by T1 hyperintense and T2 heterogeneously hypointense content, suggesting surrounding hematoma.

On MRI, a well-defined, rounded lesion showing signal void on T2-weighted images was seen within the sphenoid sinus in continuity with the cavernous segment of right ICA, surrounded by hyperintense contents on T1-weighted images, suggesting surrounding haemorrhage (Figure 3).

Diagnosis of a cavernous segment ICA aneurysm expanding into the sphenoid sinus was made.

The patient was then referred to the neurosurgery department. He was advised aneurysmal coiling with stent placement; however, patient refused the intervention and was managed conservatively.

## Discussion

Epistaxis is a common medical condition, with up to 60% of the population experiencing an episode of epistaxis in their lifetime. However, it is usually mild, with patients rarely seeking medical attention, and only a small number of patients require surgical intervention. Epistaxis is most commonly caused by dryness, local trauma, hypertension, blood dyscrasias, and rarely by sinonasal masses. ICA aneurysm with sphenoid extension causing epistaxis is very rare, and if it occurs, it is most commonly post-traumatic. Non-traumatic true ICA aneurysm presenting with epistaxis is extremely rare (1).

In most cases, epistaxis can be managed effectively by local measures such as compression of nostrils, chemical cauterisation or electrical cauterisation, vasoconstrictors, and management of the underlying condition, like control of blood pressure and correction of coagulation disorder. However, when Epistaxis is caused by aneurysm rupture, it may be massive, recurrent, or both, and can be life threatening and potentially fatal. 

Awareness about this condition is important so that relevant radiological investigation can be done to diagnose it correctly for timely management (2). Intracranial aneurysms, when unruptured, usually present with cranial nerve deficits due to compression upon the cranial nerves and can cause diplopia, gaze abnormalities, ptosis, headache, etc. When ruptured, they present with features of subarachnoid haemorrhage. The diagnosis of an aneurysm may be delayed or overlooked if presenting with epistaxis, as it is an extremely rare cause of epistaxis and hence can be life-threatening.

Cavernous internal carotid artery aneurysm accounts for less than 2% of all intracranial aneurysm (3). On routine non-contrast imaging, Cavernous Internal carotid artery neurysm may be mistaken for a sinonasal tumor or inflammatory mass, and can be biopsied, which can cause potentially catastrophic haemorrhage. So it’s imperative to recognise the imaging features of intrasphenoidal aneurysm so that further relevant radiological investigation, like CT Angiography, MR Angiography, or digital subtraction angiography, can be done for confirmation and timely management (4).

Aneurysm pathologically can be a True aneurysm or a false aneurysm, with true aneurysm being bounded by all the three layers of the vessel wall, while a false aneurysm is blood contained in perivascular tissues without a vascular wall. True aneurysm are mostly atherosclerotic and can be saccular or fusiform, while false aneurysm are mostly post-traumatic, post-surgical or post-inflammatory (1). In our case, there was no history of trauma, and the Aneurysm was considered a true aneurysm.

The cavernous segment of the ICA is closely related anatomically to the sphenoid sinus. Cadaveric studies have shown cavernous ICA to be associated with a focal bulge along the lateral wall of the sphenoid sinus, and the bone covering the sinus was less than 1mm in a remarkable number of patients, and a partial bone defect at ICA prominence has been reported in up to 23% of the general population. Pulsations from the aneurysm can also cause bone erosion due to its mechanical force, and the bone defect will allow the expansion of the aneurysm into the sinus and its subsequent rupture (5,6).

Furthermore, as there is an absence of cerebral parenchyma or meninges surrounding the aneurysm of the cavernous segment of the ICA, this surrounding anatomy can even facilitate their further growth (7). When this aneurysm ruptures, the tamponade effect of the hematoma stops the initial bleeding, but as the hematoma liquefies in the next few weeks, it will rebleed, resulting in recurrent and catastrophic bleeding with likelihood of exsanguination increasing with each subsequent episode of epistaxis (8,9). Patients presenting with epistaxis, especially when associated with cranial nerve deficits, uniocular blindness, or with a previous history of craniofacial trauma, should be suspected of having an aneurysm and evaluated radiologically to identify the aneurysm promptly, as early detection and intervention can save a patient’s life. All cases of recurrent and profuse Epistaxis should undergo a CT scan. NCCT might show hyperdense contents within the sinus with a focal defect in the sinus wall. If there is an organised hematoma surrounding the aneurysm, the sinus might be filled with heterogeneous contents, which might mimic a malignancy or an inflammatory mass lesion (like fungal infection or mucocele). CT angiography is the modality of choice and will delineate the entrapment of the ICA within the sinus wall defect and outline the aneurysm within the sinus. MRI can help in difficult cases by showing an aneurysm as a signal void on T2W sequences in continuity with the adjacent cavernous ICA and can help to differentiate the vascular structures from surrounding tissues and show associated thrombus (2).

 Haemorrhage will appear hyperintense on T1W images. A sphenoid sinus mass showing mixed T1 signal will be highly suggestive of the diagnosis, especially when there is a curvilinear laminated pattern centrally and T1 hyperintensity peripherally (4). There may be a concurrent carotid-cavernous fistula, which needs to be ruled out by looking for dilated superior ophthalmic vein and collaterals, if present (10). The presence of skull base fracture should prompt consideration of the possibility of pseudoaneurysm (Maurer’s triad is highly suggestive of skull base aneurysm and consists of history of prior facial trauma, delayed massive epistaxis, along with monocular visual disturbances) (4). If left untreated, there is a high chance of mortality with subsequent bleeds. These aneurysms can be treated by a surgical or endovascular approach. The older approach includes open Surgery with direct clipping of the aneurysm or ligation. Recently, various endovascular therapeutic options, including balloon occlusion and stent-assisted coiling, have become available. Endovascular management can be done by detachable balloons in wide-neck aneurysms or by coiling in narrow-neck aneurysms. Stent placement may also be required in some cases (4,8). 

## Conclusion

Non traumatic ICA intrasphenoidal aneurysms are infrequent causes of recurrent epistaxis and can mimic malignant or inflammatory sinus mass, which can lead to inadvertent biopsy and catastrophic bleeding. Awareness about this condition and imaging features of intrasinus aneurysm can prevent this and help in early detection and timely management by a surgical or endovascular approach.
